# Correlation Between Previous Caesarean Section and Adverse Maternal Outcomes Accordingly With Robson Classification: Systematic Review and Meta-Analysis

**DOI:** 10.3389/fmed.2021.740000

**Published:** 2022-01-10

**Authors:** Shazia Jamshed, Shuo-Chen Chien, Afifa Tanweer, Rahma-Novita Asdary, Muhammad Hardhantyo, David Greenfield, Chia-Hui Chien, Shuen-Fu Weng, Wen-Shan Jian, Usman Iqbal

**Affiliations:** ^1^Department of Pharmacy Practice, Faculty of Pharmacy, Universiti Sultan Zainal Abidin (UniSZA), Kuala Terengganu, Malaysia; ^2^Qualitative Research-Methodological Application in Health Sciences Research Group, Kulliyyah of Pharmacy, International Islamic University Malaysia, Kuantan, Malaysia; ^3^Graduate Institute of Biomedical Informatics, College of Medical Science and Technology, Taipei Medical University, Taipei, Taiwan; ^4^International Center for Health Information Technology (ICHIT), Taipei Medical University, Taipei, Taiwan; ^5^Department of Nutrition Sciences, School of Health Sciences, University of Management and Technology, Lahore, Pakistan; ^6^Masters Program in Department of Global Health & Development, College of Public Health, Taipei Medical University, Taipei, Taiwan; ^7^Graduate Program of Public Health, College of Public Health, Taipei Medical University, Taipei, Taiwan; ^8^Faculty of Health Science, Universitas Respati Yogyakarta, Depok, Indonesia; ^9^Center for Health Policy and Management, Faculty of Medicine, Public Health and Nursing Universitas Gadjah Mada, Depok, Indonesia; ^10^The Simpson Centre for Health Services Research, South Western Sydney Clinical School, University of New South Wales (UNSW) Medicine, Sydney, NSW, Australia; ^11^Linéaire Projects, Sydney, NSW, Australia; ^12^Office of Public Affairs, Taipei Medical University, Taipei, Taiwan; ^13^Division of Endocrinology and Metabolism, Department of Internal Medicine, Taipei Medical University Hospital, Taipei, Taiwan; ^14^Division of Endocrinology and Metabolism, Department of Internal Medicine, School of Medicine, College of Medicine, Taipei Medical University, Taipei, Taiwan; ^15^School of Health Care Administration, School of Gerontology Health Management, Graduate Institute of Data Science, Research Center for Artificial Intelligence in Medicine, Taipei Medical University, Taipei, Taiwan; ^16^Ph.D. Program in Depatment of Global Health & Health Security, College of Public Health, Taipei Medical University, Taipei, Taiwan

**Keywords:** previous caesarean section, adverse maternal outcomes, World Health Organisation - Robson Classification, women's health, public health practice, global health

## Abstract

**Background:** The increasing rates of Caesarean section (CS) beyond the WHO standards (10–15%) pose a significant global health concern.

**Objective:** Systematic review and meta-analysis to identify an association between CS history and maternal adverse outcomes for the subsequent pregnancy and delivery among women classified in Robson classification (RC).

**Search Strategy:** PubMed/Medline, EbscoHost, ProQuest, Embase, Web of Science, BIOSIS, MEDLINE, and Russian Science Citation Index databases were searched from 2008 to 2018.

**Selection Criteria:** Based on Robson classification, studies reporting one or more of the 14 adverse maternal outcomes were considered eligible for this review.

**Data Collection:** Study design data, interventions used, CS history, and adverse maternal outcomes were extracted.

**Main Results:** From 4,084 studies, 28 (*n* = 1,524,695 women) met the inclusion criteria. RC group 5 showed the highest proportion among deliveries followed by RC10, RC7, and RC8 (67.71, 32.27, 0.02, and 0.001%). Among adverse maternal outcomes, hysterectomy had the highest association after preterm delivery OR = 3.39 (95% CI 1.56–7.36), followed by Severe Maternal Outcomes OR = 2.95 (95% CI 1.00–8.67). We identified over one and a half million pregnant women, of whom the majority were found to belong to RC group 5.

**Conclusions:** Previous CS was observed to be associated with adverse maternal outcomes for the subsequent pregnancies. CS rates need to be monitored given the prospective risks which may occur for maternal and child health in subsequent births.

## Introduction

High rates of maternal mortality due to the common preventable causes like haemorrhage, eclampsia, and sepsis (1) call for safe procedures like Caesarean Section (CS). Although, theoretically, the procedure is intended to protect against the adverse maternal outcome, the increase in caesarean rates in low and middle-income countries has not been associated with improved perinatal outcomes (2). In addition to increased risk of neonatal and perinatal mortality in vaginal birth after caesarean (VBAC) (3), previous CS has been reported as being associated with adverse outcomes of subsequent pregnancies such as maternal mortality, blood transfusion, admission in critical care, and hysterectomy (4–6).

In 2014, the WHO proposed Robson classification for assessing, monitoring, and comparing caesarean section rates within and between healthcare facilities over time (7). The system classifies women into 10 mutually exclusive groups. There has been no previous study with a systematic review design followed by a meta-analysis that specifically discusses the history of caesarean section (repeated) with maternal and perinatal adverse outcomes by grouping the women based on the WHO classification. Previous studies have reported a relationship between the history of caesarean section and individual adverse maternal outcomes rather than pooled evidence on several maternal outcomes. The current review and meta-analysis aim at assessing women according to Robson's classification and to report pooled evidence on the impacts of previous CS on outcomes of the subsequent pregnancy.

## Methods

### Search Strategy and Selection Criteria

In this systematic review and meta-analysis, the literature was extracted by systematic search from two electronic platforms, Ovid system and Web of Science, which provided access to eight databases, including PubMed/Medline, EbscoHost, ProQuest, Embase, Web of Science, BIOSIS, MEDLINE, and Russian Science Citation Index. Studies that met all of the following criteria were regarded eligible to be included in this review: original papers reporting findings from relevant randomised controlled trials or observational study designs (cohort, cross-sectional and case-control studies) following strengthening the Reporting of Observational studies in Epidemiology (STROBE) criteria) (8), published in the English language between 2008 and 2018. Studies reporting previous CS for all participants, adverse maternal events as the outcome variable, and those providing sufficient statistical data (risk estimates) were included in this research. Only those researches were included, which were conducted on participants who had had at least one prior CS and could be classified as Robson 5,7,8,9, or 10 according to Robson classification described by the WHO (9). Studies reporting one or more of the following 14 adverse maternal outcomes were considered eligible for this review: analgesia/anaesthesia, blood transfusion, heavy bleeding, hypertension, hysterectomy, infection, maternal death, pre-eclampsia, placenta previa, preterm delivery, retained placenta, severe maternal outcomes (SMO), uterine dehiscence, and uterine rupture. The studies were excluded if they failed to report the predefined independent (CS) and outcome variables (adverse maternal outcomes), provide sufficient statistical information, were case reports, opinions, or comments on other research, were published before 2008 or after 2018, or were published in languages other than English.

The search strategy, inclusion and exclusion criteria, and extraction methods were agreed upon by all authors. Literature search and data extraction were done by one author (RN) and reviewed by another (UI). To increase sensitivity to potentially appropriate studies, free-text terms with initial keywords “caesarean section history,” “adverse maternal outcomes,” and Medical Subject Headings (MeSH) were used (Supplemenatary Table 1). In addition to the agreed-upon search strategy, citations from eligible articles were also sought for relevant literature. After title and abstract review and screening for duplicates, full texts of potentially relevant articles were examined by two independent reviewers. Variables that were extracted from each article were publication year, study setting, investigation time, study design, method of assessing the outcomes, current delivery process, indication, and current maternal outcome.

### Quality Assessment

The risk of bias (ROB) of randomised control trials was assessed with Cochrane ROB tools ver.2.0 (10). For observational designs (cohort, cross-sectional, and case-control), STROBE criteria (8) and The Newcastle-Ottawa Scale (NOS) by two independent reviewers were used for quality assessment. In assessment with NOS, a star rating system was adopted with the following classification: 0–4 stars defined as low-quality, 5–6 stars defined as medium quality, and 7–9 stars as high-quality.

### Data Analysis

The eligible studies were subjected to qualitative synthesis and statistical analysis. Epidemiological measures of risk reported in the studies, including Odds Ratio (OR), Hazard Ratio (HR), and Relative Risk (RR), were used to calculate binary outcomes and were reported as OR with 95% Confidence Interval (CI). Data on ORs extracted from studies after being grouped by adverse maternal outcomes was pooled using the random-effects model. The extracted pooled ORs for individual outcomes were combined to construct summary pooled ORs. τ2 values arising from the random-effects models were used to quantify heterogeneity among individual studies. Although the primary analysis involved all eligible studies, a secondary subgroup analysis of studies stratified based on RC was also conducted. A pooled proportion for maternal outcomes was determined for each of the RC categories using the random effects model. The statistical analysis was done using comprehensive meta-analysis and checked for accuracy. The developed protocol was prospectively registered in PROSPERO (registration number CRD42018103943).

## Results

### Study Characteristics

**Figure 1:**
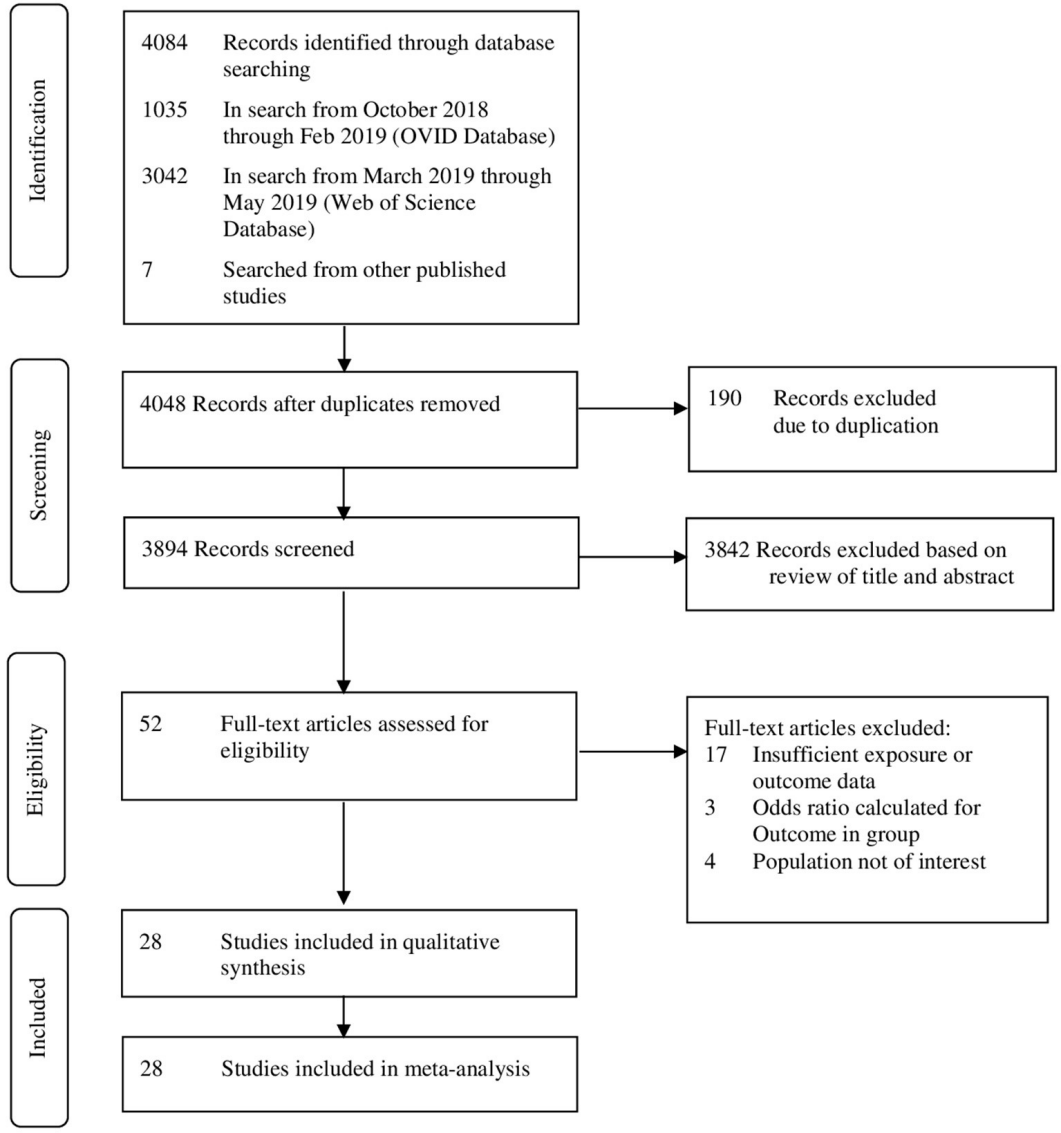
Preferred reporting items for systematic reviews and meta-analyses (PRISMA) framework for study selection.

From the initial 4,084 records, 52 articles qualified for full-text review, of which 28 were included in systematic review and meta-analysis (Figure 1). Overall, 11 prospective studies, 14 retrospective studies, one RCT, one cross-sectional, and one case-control study were included. The studies were published between 2008 and 2018 with retrospective cohorts starting from 1975. Studies reported data from six different continents. Four studies were from America (three from US and one from Canada), five studies from Australia, nine studies from Europe, seven studies from Asia, and three studies from Africa. There was also one study that covered 29 countries in Africa, Asia, Latin America, and the Middle East. Sample sizes ranged from 22 to 6,85,137 women, involving 1,524,695 women who underwent CS in the previous pregnancy (Table 1).

**Table 1:** Characteristics of studies included to find the correlation between previous caesarean section and adverse maternal outcomes.

**References**	**Design**	**Place**	**Period**	**Women**	**Data Source/Setting**	**Objective**
Asicioglu et al. (11)	Retrospective	Turkey	January 2005 and December 2010	364	Department of Maternal-Fetal Medicine of the Bakirkoy Women and Children's Teaching Hospital (Hospital A) and Sişli Etfal Teaching Hospital (Hospital B).	To investigate patient characteristics and foetal and maternal outcomes of placenta praevia and accreta
Baron et al. (12)	Retrospective	Israel	January 1, 1998, and December 31, 2011	5,635	Soroka UniversityMedical Center, Beer-Sheva, Israel	To investigate the maternal and perinatal outcomes in pregnancies associated with previous caesarean delivery and uterine scar dehiscence
Cogan et al. (13)	Retrospective	Belgium	August 2006 and March 2009	798	CHU Saint-Pierre University Hospital	To analyse, in a population of women who have a uterine scar, the maternal, foetal, and neonatal complications in relation to the mode of labour and delivery
Hammond et al. (14)	Retrospective	Australia	1984–2006	526125	Midwives Notification System (MNS) recorded in WA	To characterise changing risk factors of preterm birth in Western Australia between 1984 and 2006
Hu et al. (15)	Retrospective	China	January 2013 to December 2016	11662	International Peace Maternity and Child Health Hospital Data	To compare the perinatal outcomes of a subsequent pregnancy in women who underwent spontaneous vaginal delivery (SVD) or CS in their first delivery
Jastrow et al. (16)	Retrospective	United States	1989 and 2002	1,655	Ste-Justine Hospital Data	To evaluate obstetric outcomes in women undergoing a trial of labour (TOL) after a previous caesarean for dystocia in the second stage of labour.
Kessous et al. (17)	Retrospective	Israel	1993 and 2010	319	Soroka University Medical Center	To investigate whether vacuum extraction due to failure of labour to progress (dystocia) during the second stage in delivery following a previous caesarean section (CS) is related to increased adverse maternal and perinatal outcomes as compared with repeated CS.
Kugler et al. (18)	Retrospective	Israel	January 1988 and May 2006	1,102	Department of Obstetrics and Gynecology at the Soroka Database University Medical Center	To assess the risks of maternal and neonatal complications associated with VBAC compared to that of repeated elective caesarean section (CS) in the GMP population
Mone et al. (19)	Retrospective	Ireland	April 2010–April 2012	893	Northern Ireland Maternity System database.	To compare the characteristics of women who select elective repeat caesarean rather than a trial of labour after caesarean (TOLAC) for delivery, and to determine individual predictors for success and failure within a TOLAC group and observe differences in maternal and neonatal morbidity.
Motomura et al. (20)	Retrospective	29 countries in Africa, Asia, Latin America, and the Middle East	2010–2011	37,366	WHO Multicountry Survey on Maternal and Newborn Health (WHOMCS)	To describe the incidence, risk factors, and maternal and perinatal outcomes of uterine rupture among women with prior CS
Son et al. (21)	Retrospective	Illinois, United States	1999–2002	1,230	Caesarean Registry of the Eunice Kennedy Shriver National Institute of Child Health and Human Development Maternal-Fetal Medicine Units Network.	To compare maternal and neonatal outcomes that are associated with attempted operative vaginal delivery with those that are associated with second-stage repeat caesarean delivery without an operative vaginal delivery attempt among women who undergo a trial of labour after caesarean delivery
Stattmiller et al. (22)	Retrospective		2003–2011	685 137	Healthcare Cost and Utilization Project–Nationwide Inpatient	To evaluate the risk of adverse maternal outcomes associated with the trial of labour (TOL) after caesarean during subsequent pregnancies in the low-risk population.
Tsai and Wu (23)	Retrospective	Taiwan	January 2006 and December 2015	400	Tamshui Branch of MacKay Memorial Hospital	To reveal the world trend in VBAC and our experience of a 10-year period in a medical centre in northern Taiwan
Yao et al. (24)	Retrospective	United States	2011–2014	5,38,264	National Center for Health Statistics (NCHS), Centers for Disease Control and Prevention.	To estimate the maternal and neonatal risks associated with pregnancies that underwent TOLAC compared to those that elected for repeat caesarean delivery (RCD) in the obese population
Kabore et al. (25)	Prospective	Senegal and Mali	September 2007–October 2011	9,712	46 referral hospitals data	To assess the risks of uterine rupture, maternal and perinatal outcomes associated with a trial of labour (TOL) after one previous caesarean were compared with having an elective repeated caesarean section (ERCS) without labour in low-resource settings.
Kalisa et al. (26)	Prospective	Rwanda	June 2013 and December 2014	435	Ruhengeri district hospital medical records	To compare maternal and perinatal outcomes between ToL and elective repeat caesarean section (ERCS) at a district hospital
Al-Zirqi et al. (27)	Prospective	Norway	1 January 1999 to 30 June 2005	18,794	Medical Birth Registry of Norway (MBRN)	To determine the risk factors, percentage, and maternal and perinatal complications of uterine rupture after previous caesarean section.
Bakhshi et al. (28)	Prospective	United States	1999–2002	7,936	records from 19 academic centres	To describe the frequency of adverse maternal and neonatal outcomes at the time of repeat CD in women with a prior classical CD and compare these rates with those who had a prior low transverse CD
Belachew et al. (29)	Prospective	Sweden	1994–2006	2,58,608	Swedish Medical Birth Register	To evaluate whether women with a caesarean section at their first delivery have an increased risk of retained placenta at their second delivery
Crowther et al. (30)	Prospective	Australia	November 2002–May 2007	2,332	14 Australian Hospitals	To compare benefits and risks of a planned ERC with planned VBAC
Gilbert et al. (31)	Prospective	United States	1999–2002	22,068	The Caesarean Registry) by the Eunice Kennedy Shriver National Institute of Child Health and Human Development Maternal-Fetal Medicine Units Network	To determine outcomes, after the use of propensity score techniques, to create balanced groups according to whether a woman undergoes elective repeat caesarean delivery (ERCD) or trial of labour (TOL)
Kalok et al. (32)	Prospective	Malaysia	February 2012–September 2012	186	tertiary teaching hospital	To determine the predictive factors for a successful vaginal birth after caesarean section (VBAC) and to develop a relevant antenatal scoring system
Kok et al. (33)	Prospective	Netherland	January 2000–December 2007	19,564	Netherlands Perinatal Registry (PRN) database	To determine neonatal and short-term maternal outcomes according to the intentional mode of delivery following a caesarean delivery (CD).
Schemann et al. (34)	Prospective	New South Wales	2007–2011	61,894	NSW population databases, the Perinatal Data Collection (PDC), and the Admitted Patient Data Collection (APDC)	To determine if case mix and hospital factors explain variation in hospital rates of repeat caesarean sections and whether these rates are associated with maternal and neonatal morbidity.
Studsgaard et al. (35)	Prospective	Denmark	March 2003–December 2010	1,783	Danish university hospital	To compare outcomes with the trial of labour after caesarean (TOLAC) or elective repeat caesarean delivery on maternal request (ERCD-MR)
Crowther et al. (30)	RCT	Australia	November 2002–May 2007	22	14 Australian Hospitals	To compare benefits and risks of a planned ERC with planned VBAC
Litorp et al. (36)	Cross-sectional	Tanzania	February–June 2012	2,478	Uhimbili National Hospital in Dar es Salaam	To investigate if multiparous individuals who had undergone a previous caesarean delivery experienced an increased risk of severe maternal outcomes or adverse perinatal outcomes compared with multiparous individuals who had undergone previous vaginal deliveries
Homer et al. (37)	Case-control study	United Kingdom	February 2005 and February 2006	923	UK Obstetric Surveillance System	To examine whether the TGCS could be extended in a novel way to classify who required a peripartum hysterectomy

### Link Between Previous Caesarean Section and Adverse Maternal Outcomes

The most common adverse maternal outcomes reported were heavy bleeding (reported in 15 studies) and uterine rupture (reported in 12 studies). Analgesia/Anaesthesia administration (98.21% in CS group, 93.84% in VBAC group), Infection (16.28% in CS group, 8.50% in VBAC group), and heavy bleeding (5.68% in CS group, 3,84% in VBAC group) were among the highest reported events (Supplementary Table 2).

**Figure 2:**
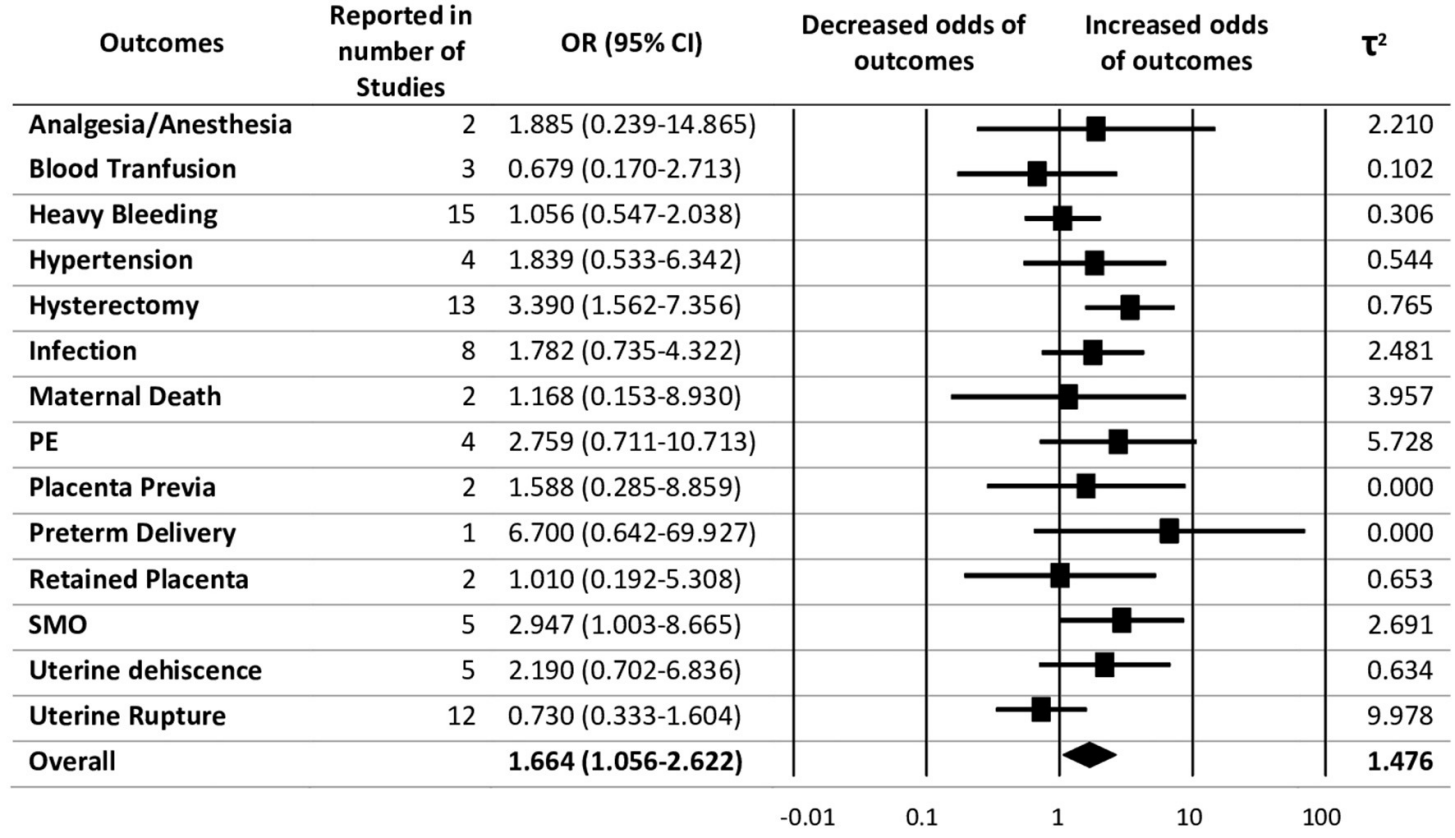
Summary of pooled odds ratios (ORs) for the correlation between previous caesarean section (CS) with maternal adverse outcomes.

The pooled evidence for risk of adverse maternal outcomes with previous CS has been shown in Figure 2. Random-effects analysis showed an association between previous CS with adverse maternal outcomes with an overall pooled effect size of 1.66 (95% CI 1.06–2.62) and heterogeneity as τ*2* = 1.48. Of the adverse maternal outcomes, hysterectomy was found to have the highest association with previous CS after preterm delivery with OR = 3.39 (95% CI 1.56–7.36), followed by severe maternal outcomes with OR = 2.95 (95% CI 1.00–8.67).

### Link Between Previous Caesarean Section and Adverse Maternal Outcomes Based on Robson Classification

**Figure 3:**
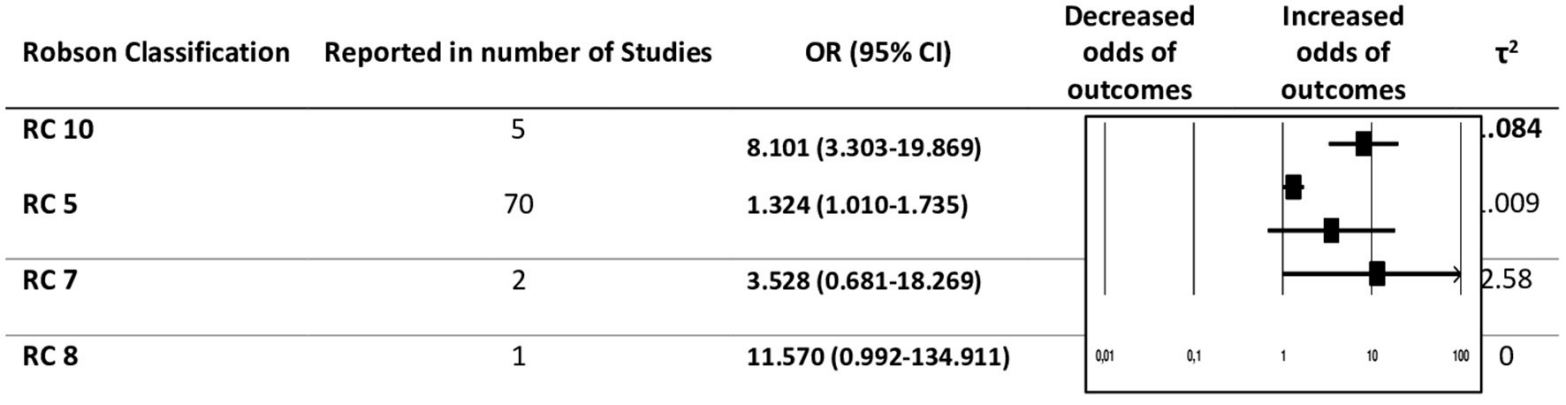
Robson subgroup analysis with summary of pooled odds ratios (ORs).

The studies which qualified for the final analysis reported women belonging to four groups of Robson Classification (RC5, RC7, RC8, and RC10). RC5 was the most commonly reported group in the selected studies. The outcomes reported in RC5 varied into 13 different maternal adverse outcomes. Despite being the most commonly reported class, the overall pooled effect of RC5 with adverse maternal outcomes was found to be 1.32 (95% CI 1.01–1.74) (Figure 3).

### Publication Bias

**Figure 4:**
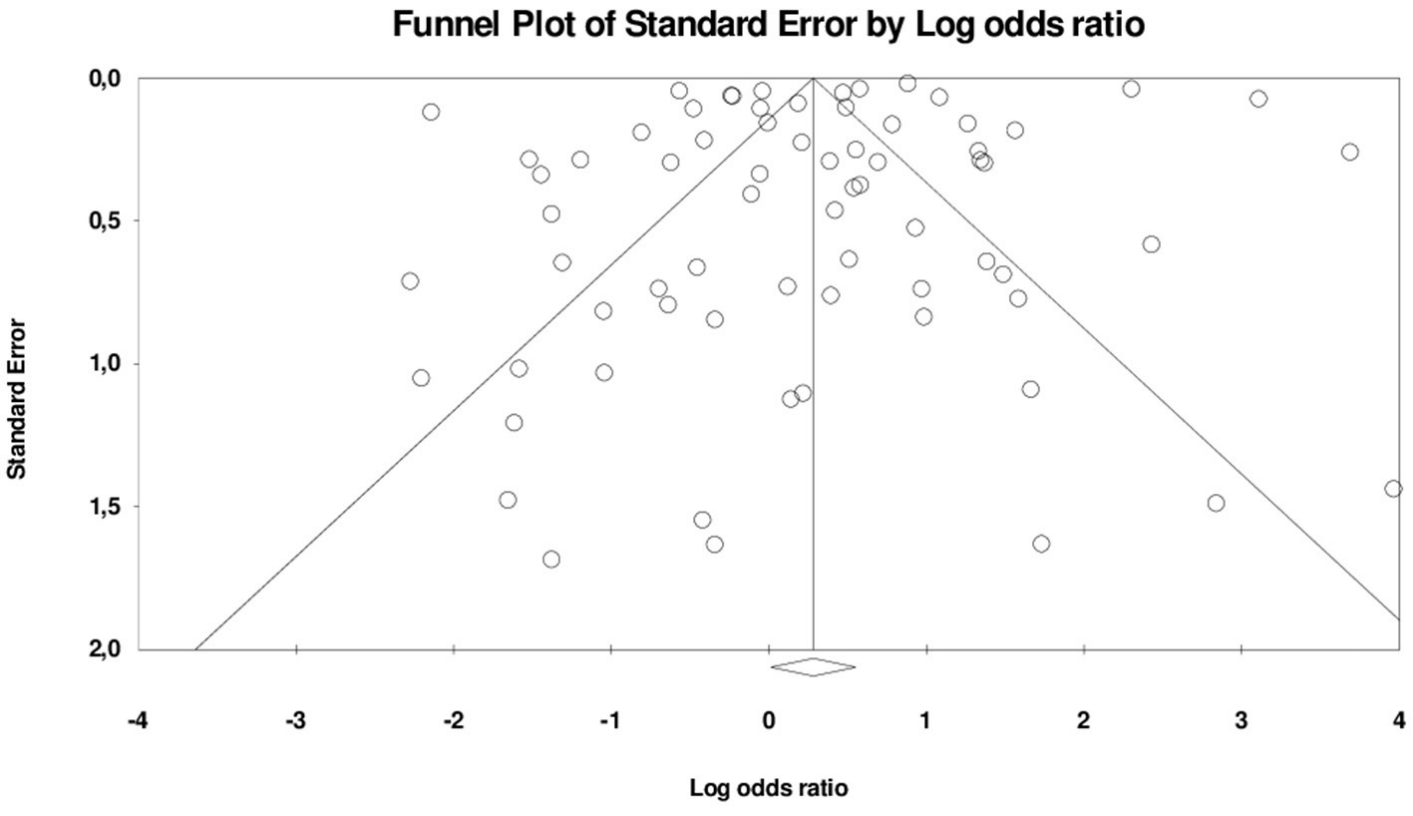
Funnel plot RC 5 with adverse maternal outcomes.

Among the four subgroups of Robson Classification, only RC5, as reported in 70 different studies, was regarded as eligible for assessment of publication bias. Assessment for publication bias was not performed for other groups (≤5 studies). The funnel plot and Egger's test (Figure 4) showed no evidence of a significant small-study effect in the analyses between previous CS and adverse maternal outcomes for subgroup RC5 (*p* = 0.20).

### Meta-Regression for Exploring Between-Study Heterogeneity

To explore the sources of study heterogeneity, meta-regression with covariates publication year, countries, study design, and the sample size was carried out. As individual-level data were unavailable, we used aggregate data for this purpose. The result showed there is no between-study heterogeneity (*p* = 0.57).

### Quality Appraisal and Risk of Bias Assessment

Of the 27 observational studies assessed by the star rating system of NOS, one was regarded to be of low quality, 16 as medium quality, and 10 as high quality. One randomised control trial assessed by using Cochrane ROB tools (version 2.0) showed a low risk of bias (Supplementary Figure 1).

## Discussion

In this meta-analysis of the data of 1,524,695 individuals from diversified regions around the world, the previous CS was found to be associated with adverse maternal outcomes in subsequent pregnancy and childbirth. A two-way link between a history of CS and adverse maternal outcomes was observed. The odds of experiencing adverse outcomes for women who experienced repeat-CS was 1.61-fold the odds of someone who went through the VBAC.

Among the adverse outcomes studied, hysterectomy was one of the most common events. The odds of hysterectomy for women who experienced repeat-CS were found to be 3.390-fold the odds of someone who went through VBAC. This result is in accordance with a previous study that showed elective repeated caesarean delivery might be associated with a higher risk of hysterectomy and neonatal respiratory problems (38). Hysterectomy as a life-saving intervention is frequently needed for patients with previous CS, especially when the excessive blood loss treatment intervention has been done. Since the women in the repeat CS group had a higher rate of hysterectomy, this strengthens the association of previous CS with the adverse maternal outcomes that occur during the subsequent birth. Cephalic presentation in the Robson Classification group 5 is not the leading cause, but the previous CS has a significant association with the hysterectomy event. The underlying factors associated with the increased likelihood of hysterectomy are adherent placenta, placenta previa (39–41), postpartum haemorrhage (40), and previous CS (41, 42).

The odds of severe maternal outcomes for women who experienced repeat-CS were 3-fold the odds of someone who went through VBAC. There are two previous studies that have suggested that maternal near-miss (MNM) events and maternal deaths should be coupled to reflect SMO, providing a more robust variable for study. Previous caesarean delivery in relation to MNM and SMO has been explored and found that individuals with previous caesarean deliveries have an increased risk of MNM and SMO (43, 44). In this study, we excluded maternal death from the SMO group after introducing a separate maternal death outcome category. Interestingly, SMO was only reported by five different studies in Robson classification group 5. Women who experienced at least one previous CS with the cephalic presentation were more likely to have severe maternal outcomes in the subsequent pregnancy and childbirth. An enhanced probability of SMO/MNM has been reported to be associated with previous CS, high parity, and age (43).

We found that women who showed Pre-eclampsia outcomes were three times more likely to experience repeat-CS than those VBAC. Pre-eclampsia might be related to gravidity. Women giving birth to their fourth child through CS can be three times more likely to experience pre-eclampsia compared to gravida 1 (45). This study also revealed that women who have uterine dehiscence as the outcome are more likely to experience repeat-CS than those who had VBAC. Uterine dehiscence is a disruption of the uterine muscle with intact serosa (28). Uterine dehiscence and admission to the intensive care unit were more common in women with a prior classical CS. As a result of CS operation, late scar dehiscence may occur, which may lead to uterine rupture in a subsequent pregnancy (46). The uterine scar from previous CS is prone to be damaged due to both enlarged uterine and uterine contraction. The odds of hypertension for women who experienced repeat-CS were two-fold than those who went through VBAC. From three studies reporting hypertension as the adverse outcomes in RC group 5, one study has a diverse effect size. The effect size of this outcome reported in RC 10, the odds of women who has previous CS with premature birth are five-fold the odds of a woman who went through VBAC (OR = 5.16; 95% CI 4.52–5.89). Women with chronic hypertension are more likely to have various issues, including superimposed Pre-eclampsia and CS (47). It is also probable that other threat variables for chronic hypertension, including obesity and metabolism, will increase (48). Therefore, the number of women having a pregnancy with established chronic hypertension can result in an increasing rate of CS.

The current analysis showed that repeated CS was associated with a higher risk (about two-fold) of analgesia/anaesthesia administration than those who had VBAC. These findings are consistent with the previous study reported that those mothers who were treated with epidural analgesia during labour have higher chances of undergoing CS because of foetal distress (49). With the worldwide rise in the frequency of CS, the incidence of infection is anticipated to rise in conjunction, hence its clinical significance. Women undergoing repeat CS were twice likely to become infected than those undergoing VBAC. This finding is supported by a previous study that reported prior CS as one of the infection risk factors apart from maternal age, obesity, rural (as opposed to urban) dwelling, pre-gestational disease Mellitus, and pre-operative maternal condition (50). Post-CS infection usually results from a bacterial infection on the surgical site of the incision. Women with vaginal deliveries are less likely to get this infection. This study showed that infection cases were reported by eight different studies, even in women belonging to Robson Classification group 5. There was, however, no report regarding infection of women with previous CS with multiple pregnancies, oblique lie, breech presentation, or preterm pregnancy in the subsequent pregnancy and birth. There is evidence available to suggest the long term-effect of CS. With the rate of previous CS rising from 12 to 38% in over a decade, the placenta praevia frequency has increased. The occurrence of placenta praevia as the consequence where the lower uterine segment is scarred due to previous CS was reported by several studies (51, 52). In the current study, the odds of placenta praevia for women who experienced repeat-CS are almost two-fold the odds of a woman who went through VBAC. This outcome is associated with abnormalities in the endometrium triggered by prior scarring due to previous CS. In addition, in pregnancies with placenta previa and accreta, maternal age gives a significant contribution. Also, higher maternal age impairs ordinary placental growth as intramyometrial and endometrial arteries degrade with advanced maternal age (11).

In contrast to all other maternal outcomes, previous CS was found to be protective for blood transfusion and uterine rupture. Following a prior primary caesarean, a higher risk of blood transfusion has been reported to be associated with attempting VBAC compared with repeated CS (53). However, evidence also suggests opposing findings, suggesting the risk of blood transfusion is high in CS. Preoperative anaemia, high parity, and serious blood loss during operation lead considerably to the need for blood transfusion in patients experiencing CS (54, 55). These unexpected findings are probably because of underreporting in the databases of each study leading to an underestimation of the effect.

We also found that women who have repeated CS were about 27% less likely to have uterine rupture as compared to those who had VBAC. According to the American College of Obstetricians and Gynecologists (ACOG), in a previous caesarean with a low transverse incision, the risk of uterine rupture in a vaginal delivery is about 1 chance in 500. Smith et al. published that women with failed VBAC are at higher risk of uterine rupture and perinatal death (56). Another study by Hochler et al. concluded a 0.3% risk of uterine rupture, and two cases ended in hysterectomy during their retrospective study to evaluate the safety of trial of labour after caesarean delivery in multiparous women (57). In this analysis, all of 12 studies reporting uterine rupture were in the RC group 5. This could contribute to some women being misclassified in the 10 groups because some of the studies excluded women with several comorbidities such as twin gestation and oblique lie.

There were no distinctions in the results between the repeated CS and the VBAC for preterm delivery, heavy bleeding (OR = 1.06; 95% CI.55–2.04), retained placenta (OR = 1.01; 95% CI.19–5.31), and maternal death (OR = 1.17; 95% CI.15–8.93). We could not report that preterm delivery has a very high association with the previous CS because, among the studies included in this review, there was only one study reporting preterm delivery as the outcome of the current birth after previous CS. Risk factors related to prior and existing obstetric problems (earlier premature birth, prior caesarean delivery, pre-eclampsia, and antepartum haemorrhage) were the most important predictors of premature birth and negative labour onset (14).

Even though this study resulted in no association between previous CS with heavy bleeding, retained placenta, and maternal death, the thorough clinical analysis identified retained placenta and co-occurring placenta praevia as the most common cause of haemorrhage (39). These factors were especially important for those women whose CS earlier. In keeping with guidelines by the Royal College of Obstetricians and Gynaecologists, the vast majority of women with previous CS had an antenatal ultrasound for placental location. Almost all women with retained placenta-indicated haemorrhage had previously delivered by CS (58). While it is recognised that the final diagnosis of the retained placenta can only be made during surgery, the occurrence of unreported instances shows the need for changes in antenatal identification.

The risk factors of the maternal death reported by two studies were postpartum haemorrhage, uterine rupture, pre-eclampsia/eclampsia, postpartum infection, and other obstetric complications (20, 25). Maternal death should be prevented by operative procedures, such as CS, given the changing birth patterns with higher CS rates in most countries. However, the increase in caesarean rates was not associated with improved outcomes, regardless of whether the starting caesarean rate was already high (2). The healthcare professional can provide either elective or primary CS. Meanwhile, the overall women in these two studies were in the RC group 5, which means that all the women with previous CS were having a cephalic presentation. Unfortunately, Robson's classification did not subgroup women into more specific classification so that we can understand the main cause of maternal death.

Implementation of the Robson Classification may have limitations, mainly related to the availability and validity of information on the onset of labour and duration of pregnancy at delivery. One study proposed subdivision for the 10-group classification system according to augmentation or no augmentation, spontaneous/induced/CS before labour, with/without a previous uterine scar, previous or no previous vaginal delivery, and one or more than one previous scar (59). These subdivision systems for the group of women match with the group we use in this study (Robson classification group 5, 7, 8, 9, 10). Another study showed that groups 6–10 were smaller groups with high percentages of CS due to unavoidable obstetric indications (60). Therefore, group subdivision for the Robson Classification group is necessary. When compared with other studies internationally, almost all studies conveyed comparable results in groups 6–10. Using subgroup assessment for women with special needs and comorbidities or examining outcomes other than CS, especially hysterectomy, as part of a new system to monitor is recommended.

In summary, previous CS suggests higher risk and poorer clinical outcomes for women across a range of factors during and post pregnancy and birth. Conversely, and somewhat unexpectedly, other outcomes were not impacted or lowered. Hence, clinical impact and outcomes from repeated CS remain diverse and impacted by individual factors. Therefore, we recommend that health professionals must counsel women demanding a repeat CS in light of the findings of this meta-analysis and synthesis.

The current review and analysis have some methodological limitations, including that qualitative synthesis could be subjective. The data was extracted using only two databases and did not include unpublished work on the subject matter. Study heterogeneity may have affected the reliability of results. After we performed meta-regression that yielded the population size, year, and study design have no contribution to between-study heterogeneity, we did not perform the further analysis. We suggest future researchers explore the implications of elective CS, emergency CS and trial of labour on adverse maternal outcomes.

## Conclusion

While recognising the benefits that CS can bring to reduce maternal mortality and perinatal outcomes, it needs to be recognised that these are yet to be realised in low- and middle-income countries. Additionally, there are increased risks for subsequent pregnancies, for both mother and child.

## Data Availability Statement

The datasets presented in this study can be found in online repositories. The names of the repository/repositories and accession number(s) can be found in the article/Supplementary Material.

## Author Contributions

R-NA and UI designed the study. S-FW and W-SJ provided important feedback on the proposed study design. S-CC, AT, R-NA, and MH conducted the systematic literature search and quality assessment. AT, R-NA, and MH conducted the meta-analyses and the results were interpreted by all authors (SJ, S-CC, AT, R-NA, MH, DG, C-HC, S-FW, W-SJ, and UI). SJ and AT drafted the initial manuscript, which was thoroughly reviewed for important intellectual content and revised by all authors (S-CC, R-NA, MH, DG, C-HC, S-FW, W-SJ, and UI). All authors approved the final manuscript as submitted and agreed to be accountable for all aspects of the work.

## Funding

This work was supported in part by the Ministry of Science of Technology (MOST) and the project numbers are MOST110-2221-E-038-020 and MOST110-2221-E-038-007.

## Conflict of Interest

The authors declare that the research was conducted in the absence of any commercial or financial relationships that could be construed as a potential conflict of interest.

## Publisher's Note

All claims expressed in this article are solely those of the authors and do not necessarily represent those of their affiliated organizations, or those of the publisher, the editors and the reviewers. Any product that may be evaluated in this article, or claim that may be made by its manufacturer, is not guaranteed or endorsed by the publisher.

## References

[1] Say L Chou D Gemmill A Tuncalp O Moller AB Daniels J . Global causes of maternal death: a WHO systematic analysis. Lancet Global health. (2014) 2:e323–33. 10.1016/S2214-109X(14)70227-X 25103301

[2] Zhao Y Zhang J Zamora J Vogel JP Souza JP Jayaratne K . Increases in caesarean delivery rates and change of perinatal outcomes in low- and middle-income countries: a hospital-level analysis of two WHO surveys. Paediatr Perinat Epidemiol. (2017) 31:251–62. 10.1111/ppe.12363 28474743

[3] Moraitis AA Oliver-Williams C Wood AM Fleming M Pell JP Smith G. Previous caesarean delivery and the risk of unexplained stillbirth: retrospective cohort study and meta-analysis. BJOG. (2015) 122:1467–74. 10.1111/1471-0528.13461 26033155

[4] Cheng KK Lee MM. Rising incidence of morbidly adherent placenta and its association with previous caesarean section: a 15-year analysis in a tertiary hospital in Hong Kong. Hong Kong Med J. (2015) 21:511–7. 10.12809/hkmj154599 26554269

[5] Karagiozova J Ivanov S Masseva A Frandeva B Ibriam I. Location of the placenta in pregnancy with previous caesarean section. Akusherstvo i ginekologiia. (2014) 53 Suppl 2:26–8. 25510049

[6] Sholapurkar SL. Increased incidence of placenta praevia and accreta with previous caesareans–a hypothesis for causation. J Obstet Gynaecol. (2013) 33:806–9. 10.3109/01443615.2013.823388 24219718

[7] WHO HRP. WHO Statement on Caesarean Section Rates. Washington, DC: World Health Organization (2015).

[8] STROBE Statement (2009). Available online at: https://www.strobe-statement.org/index.php?id=strobe-home

[9] WHO. Robson Classification Interpretation Manual. Switzerland: WHO Press (2017).

[10] Higgins J Savović J Page M. Revised Cochrane risk of bias tool for randomized trials (RoB 2.0). Version (2016). Available online at: https://www.unisa.edu.au/contentassets/72bf75606a2b4abcaf7f17404af374ad/rob2-0_indiv_main_guidance.pdf

[11] Asicioglu O Sahbaz A Gungorduk K Yildirim G Asicioglu BB Ulker V. Maternal and perinatal outcomes in women with placenta praevia and accreta in teaching hospitals in Western Turkey. J Obstet Gynaecol. (2014) 34:462–6. 10.3109/01443615.2014.902040 24734898

[12] Baron J Weintraub AY Eshkoli T Hershkovitz R. Sheiner E. The consequences of previous uterine scar dehiscence and cesarean delivery on subsequent births. Int J Gynaecol Obstet. (2014) 126:120–2. 10.1016/j.ijgo.2014.02.022 24825500

[13] Cogan A Barlow P Benali N Murillo D Manigart Y Belhomme J . An audit about labour induction, using prostaglandin, in women with a scarred uterus. Arch Gynecol Obstet. (2012) 286:1399–406. 10.1007/s00404-012-2481-5 22836816

[14] Hammond G Langridge A Leonard H Hagan R Jacoby P DeKlerk N . Changes in risk factors for preterm birth in Western Australia 1984-2006. BJOG. (2013) 120:1051–60. 10.1111/1471-0528.12188 23639083

[15] Hu H-T Xu J-J Lin J Li C Wu Y-T Sheng J-Z . Association between first caesarean delivery and adverse outcomes in subsequent pregnancy: a retrospective cohort study. BMC Pregnancy Childbirth. (2018) 18:273. 10.1186/s12884-018-1895-x 29954355 PMC6027796

[16] Jastrow N Demers S Gauthier RJ Chaillet N Brassard N. Bujold E. Adverse obstetric outcomes in women with previous cesarean for dystocia in second stage of labor. Am J Perinatol. (2013) 30:173–8. 10.1055/s-0032-1322515 22836821

[17] Kessous R Tirosh D Weintraub AY Benshalom-Tirosh N Sergienko R. Sheiner E . Second stage disorders in patients following a previous cesarean section: vacuum versus repeated cesarean section. Arch Gynecol Obstet. (2013) 287:1075–9. 10.1007/s00404-012-2688-5 23274791

[18] Kugler E Shoham-Vardi I Burstien E Mazor M. Hershkovitz R. The safety of a trial of labor after cesarean section in a grandmultiparous population. Arch Gynecol Obstet. (2008) 277:339–44. 10.1007/s00404-007-0490-6 17957377

[19] Mone F Harrity C Toner B Mcnally A Adams B. Currie A. Predicting why women have elective repeat cesarean deliveries and predictors of successful vaginal birth after cesarean. Int J Gynaecol Obstet. (2014) 126:67–9. 10.1016/j.ijgo.2013.12.013 24731437

[20] Motomura K Ganchimeg T Nagata C Ota E Vogel JP Betran AP . Incidence and outcomes of uterine rupture among women with prior caesarean section: WHO multicountry survey on maternal and newborn health. Sci Rep. (2017) 7:44093. 10.1038/srep44093 28281576 PMC5345021

[21] Son M Roy A Grobman WA. Attempted operative vaginal delivery vs repeat cesarean in the second stage among women undergoing a trial of labor after cesarean delivery. Am J Obstet Gynecol. (2017) 216:407.e1–e5. 10.1016/j.ajog.2017.01.013 28153660

[22] Stattmiller S Lavecchia M Czuzoj-Shulman N Spence A Abenhaim HA. Trial of labor after cesarean in the low-risk obstetric population: a retrospective nationwide cohort study. J Perinatol. (2016) 36:808–13. 10.1038/jp.2016.36 27253892

[23] Tsai H-T. Wu C-H. Vaginal birth after cesarean section—The world trend and local experience in Taiwan. Taiwan J Obstet Gynecol. (2017) 56:41–5. 10.1016/j.tjog.2016.03.007 28254224

[24] Yao R Crimmins SD Contag SA Kopelman JN. Goetzinger KR. Adverse perinatal outcomes associated with trial of labor after cesarean section at term in pregnancies complicated by maternal obesity. J Matern Fetal Neonatal Med. (2019) 32:1256–61. 10.1080/14767058.2017.1404023 29172787

[25] Kabore C Chaillet N Kouanda S Bujold E Traore M Dumont A. Maternal and perinatal outcomes associated with a trial of labour after previous caesarean section in sub-Saharan countries. BJOG. (2016) 123:2147–55. 10.1111/1471-0528.13615 26374554

[26] Kalisa R Rulisa S van Roosmalen J van den Akker T. Maternal and perinatal outcome after previous caesarean section in rural Rwanda. BMC Pregnancy Childbirth. (2017) 17:272. 10.1186/s12884-017-1467-5 28841838 PMC5574082

[27] Al-Zirqi I Stray-Pedersen B Forsén L. Vangen S. Uterine rupture after previous caesarean section. BJOG. (2010) 117:809–20. 10.1111/j.1471-0528.2010.02533.x 20236103

[28] Bakhshi T Landon MB Lai YL Spong CY Rouse DJ Leveno KJ . Maternal and neonatal outcomes of repeat cesarean delivery in women with a prior classical versus low transverse uterine incision. Am J Perinatol. (2010) 27:791–5. 10.1055/s-0030-1254238 20458666 PMC2955172

[29] Belachew J Cnattingius S Mulic-Lutvica A Eurenius K Axelsson O. Wikström A-K. Risk of retained placenta in women previously delivered by caesarean section: a population-based cohort study. BJOG. (2014) 121:224–9. 10.1111/1471-0528.12444 24044730

[30] Crowther CA Dodd JM Hiller JE Haslam RR Robinson JS Birth After Caesarean Study Group. Planned vaginal birth or elective repeat caesarean: patient preference restricted cohort with nested randomised trial. PLoS Med. (2012) 9:e1001192. 10.1371/journal.pmed.1001192 22427749 PMC3302845

[31] Gilbert SA Grobman WA Landon MB Spong CY Rouse DJ Leveno KJ . Elective repeat cesarean delivery compared with spontaneous trial of labor after a prior cesarean delivery: a propensity score analysis. Am J Obstet Gynecol. (2012) 206:311.e1–e9. 10.1016/j.ajog.2012.02.002 22464069 PMC3337034

[32] Kalok A Zabil SA Jamil MA Lim PS Shafiee MN Kampan N . Antenatal scoring system in predicting the success of planned vaginal birth following one previous caesarean section. J Obstet Gynaecol. (2018) 38:339–43. 10.1080/01443615.2017.1355896 29017359

[33] Kok N Ruiter L Lindeboom R De Groot C Pajkrt E Mol B . Elective repeat cesarean delivery compared with trial of labor after a prior cesarean delivery: a propensity score analysis. Eur J Obstet Gynecol Reprod Biol. (2015) 195:214–8. 10.1016/j.ejogrb.2015.09.011 26599733

[34] Schemann K Patterson JA Nippita TA Ford JB. Roberts CL. Variation in hospital caesarean section rates for women with at least one previous caesarean section: a population based cohort study. BMC Pregnancy Childbirth. (2015) 15:179. 10.1186/s12884-015-0609-x 26285692 PMC4545707

[35] Studsgaard A Skorstengaard M Glavind J Hvidman L. Uldbjerg N. Trial of labor compared to repeat cesarean section in women with no other risk factors than a prior cesarean delivery. Acta Obstet Gynecol Scand. (2013) 92:1256–63. 10.1111/aogs.12240 23962339

[36] Litorp H Rööst M Kidanto HL Nyström L. Essén B. The effects of previous cesarean deliveries on severe maternal and adverse perinatal outcomes at a university hospital in Tanzania. Int J Gynaecol Obstet. (2016) 133:183–7. 10.1016/j.ijgo.2015.10.009 26868073

[37] Homer CS Kurinczuk JJ Spark P Brocklehurst P. Knight M. A novel use of a classification system to audit severe maternal morbidity. Midwifery. (2010) 26:532–6. 10.1016/j.midw.2010.03.010 20691518

[38] Yang YZ Ye XP Sun XX. Maternal and neonatal morbidity: repeat cesarean versus a trial of labour after previous cesarean delivery. Clin Invest Med. (2017) 40:E135–E45. 10.25011/cim.v40i3.28393 28653615

[39] Campbell SM Corcoran P Manning E Greene RA Irish Maternal Morbidity Advisory Group. Peripartum hysterectomy incidence, risk factors and clinical characteristics in Ireland. Eur J Obstet Gynecol Reprod Biol. (2016) 207:56–61. 10.1016/j.ejogrb.2016.10.008 27825028

[40] Cheng HC Pelecanos A Sekar R. Review of peripartum hysterectomy rates at a tertiary Australian hospital. Aust N Z J Obstet Gynaecol. (2016) 56:614–8. 10.1111/ajo.12519 27535339

[41] D'Arpe S Franceschetti S Corosu R Palaia I Di Donato V Perniola G . Emergency peripartum hysterectomy in a tertiary teaching hospital: a 14-year review. Arch Gynecol Obstet. (2015) 291:841–7. 10.1007/s00404-014-3487-y 25253416

[42] Higgins MF Monteith C Foley M O'Herlihy C. Real increasing incidence of hysterectomy for placenta accreta following previous caesarean section. Eur J Obstet Gynecol Reprod Biol. (2013) 171:54–6. 10.1016/j.ejogrb.2013.08.030 24157231

[43] Souza JP Cecatti JG Faundes A Morais SS Villar J Carroli G . Maternal near miss and maternal death in the World Health Organization's 2005 global survey on maternal and perinatal health. Bull World Health Organ. (2010) 88:113–9. 10.2471/BLT.08.057828 20428368 PMC2814475

[44] Souza JP Cecatti JG Haddad SM Parpinelli MA Costa ML Katz L . The WHO maternal near-miss approach and the maternal severity index model (MSI): tools for assessing the management of severe maternal morbidity. PLoS ONE. (2012) 7:e44129. 10.1371/journal.pone.0044129 22952897 PMC3430678

[45] Al Rowaily MA Alsalem FA Abolfotouh MA. Cesarean section in a high-parity community in Saudi Arabia: clinical indications and obstetric outcomes. BMC Pregnancy Childbirth. (2014) 14:92. 10.1186/1471-2393-14-92 24575731 PMC3941573

[46] Klemm P Koehler C Mangler M Schneider U Schneider A. Laparoscopic and vaginal repair of uterine scar dehiscence following cesarean section as detected by ultrasound. J Perinat Med. (2005) 33:324–31. 10.1515/JPM.2005.058 16207118

[47] Guedes-Martins L. Chronic hypertension and pregnancy. Adv Exp Med Biol. (2017) 956:395–407. 10.1007/5584_2016_81 27722962

[48] Bateman BT Bansil P Hernandez-Diaz S Mhyre JM Callaghan WM Kuklina EV. Prevalence, trends, and outcomes of chronic hypertension: a nationwide sample of delivery admissions. Am J Obstetr Gynecol. (2012) 206:134.e1–8. 10.1016/j.ajog.2011.10.878 22177190 PMC4103984

[49] Anim-Somuah M Smyth RM Jones L. Epidural versus non-epidural or no analgesia in labour. Cochrane Database Syst Rev. (2011) 5:Cd000331. 10.1002/14651858.CD000331.pub3 22161362

[50] Zuarez-Easton S Zafran N Garmi G Salim R. Postcesarean wound infection: prevalence, impact, prevention, and management challenges. Int J Womens Health. (2017) 9:81–8. 10.2147/IJWH.S98876 28255256 PMC5322852

[51] Asicioglu O Gungorduk K Yildirim G Asicioglu BB Gungorduk OC Ark C . Second-stage vs first-stage caesarean delivery: Comparison of maternal and perinatal outcomes. J Obstet Gynaecol. (2014) 34:598–604. 10.3109/01443615.2014.920790 24911878

[52] Memon S Kumari K Yasmin H Bhutta S. Is it possible to reduce rates of placenta praevia? J Pak Med Assoc. (2010) 60:566−9. Available online at: https://ecommons.aku.edu/pakistan_fhs_mc_women_childhealth_obstet_gynaecol/9/20578609

[53] Pont S Austin K Ibiebele I Torvaldsen S Patterson J Ford J. Blood transfusion following intended vaginal birth after cesarean versus elective repeat cesarean section in women with a prior primary cesarean: a population-based record linkage study. Obstetr Anesthesia Digest. (2019) 39:189–90. 10.1097/01.aoa.0000603680.30791.46 30431154

[54] Akinlusi FM Rabiu KA Durojaiye IA Adewunmi AA Ottun TA Oshodi YA. Caesarean delivery-related blood transfusion: correlates in a tertiary hospital in Southwest Nigeria. BMC Pregnancy Childbirth. (2018) 18:24. 10.1186/s12884-017-1643-7 29320992 PMC5764010

[55] Eyelade O Adesina O Adewole I Adebowale S. Blood transfusion requirement during caesarean delivery: risk factors. Ann Ibadan Postgraduate Med. (2015) 13:29–35. Available online at: https://www.ajol.info/index.php/aipm/article/view/127742 26807084 PMC4715370

[56] Smith GC White IR Pell JP Dobbie R. Predicting cesarean section and uterine rupture among women attempting vaginal birth after prior cesarean section. PLoS Med. (2005) 2:e252. 10.1371/journal.pmed.0020252 16146414 PMC1201366

[57] Hochler H Yaffe H Schwed P Mankuta D. Safety of trial of labor after cesarean delivery in grandmultiparous women. Obstet Gynecol. (2014) 123:304–8. 10.1097/AOG.0000000000000082 24402589

[58] Jauniaux E Alfirevic Z Bhide A Belfort M Burton G Collins S . Placenta Praevia, Placenta Praevia Accreta and Vasa Praevia: Diagnosis and Management. London: RCOG. 2018. p. 1-26. 10.1111/1471-0528.15306

[59] Betran AP Vindevoghel N Souza JP Gulmezoglu AM Torloni MR. A systematic review of the Robson classification for caesarean section: what works, doesn't work and how to improve it. PLoS ONE. (2014) 9:e97769. 10.1371/journal.pone.0097769 24892928 PMC4043665

[60] Kazmi T Sarva Saiseema V Khan S. Analysis of cesarean section rate-according to Robson's 10-group classification. Oman Med J. (2012) 27:415. 10.5001/omj.2012.102 23074555 PMC3472574

