# Supplementary material for: Correlation Between Previous Caesarean Section and Adverse Maternal Outcomes Accordingly With Robson Classification: Systematic Review and Meta-Analysis
Source: Front Med (Lausanne). 2022 Jan 10;8:740000. doi: 10.3389/fmed.2021.740000 (PMC8795992; doi:10.3389/fmed.2021.740000)
Supplement: Supplementary file 1 [file Data_Sheet_1.DOCX]

Appendix

**Contents**

STable 1. Characteristic of Studies Grouped by Maternal Adverse Outcomes ………………………………………………………………………..2

STable 2. Subgroup Analysis The Correlation of Previous Caesarean Section and Adverse Maternal Outcomes …………………………………..7

STable 3 Research Variables Operational Definition - Correlation between Previous CS and Adverse Maternal Outcomes ……………………...10

STable 4 Methodological quality assessment of the observational studies using the Newcastle Ottawa Scale ……………………………………..11

Figure A. Risk of bias assessed with Cochrane ROB tools ver.2.0 for Experimental Studies ………………………………………….12

STable 1

Characteristic of Studies Grouped by Maternal Adverse Outcomes

| Outcomes | Studies | RC | OR | Lower Limit | Upper limit | Weight (%) |
| --- | --- | --- | --- | --- | --- | --- |
| Analgesia/Anesthesia | Crowther, 2012 | Rb5 | 3.805 | 2.287 | 6.328 | 74.083 |
|  | Crowther, 2012 RCT | Rb5 | 0.253 | 0.009 | 6.937 | 25.917 |
|  | Overall |  | *1.885* | *0.239* | *14.865* |  |
| Blood Transfusion | Kessous, 2013 | Rb5 | 0.900 | 0.402 | 2.012 | 31.215 |
|  | Kok, 2015 | Rb5 | 0.448 | 0.306 | 0.656 | 33.995 |
|  | Yao, 2017 | Rb5 | 0.792 | 0.697 | 0.900 | 34.789 |
|  | Overall |  | *0.679* | *0.170* | *2.713* |  |
| Heavy Bleeding | Belachew, 2014 | Rb5 | 1.610 | 1.444 | 1.795 | 7.848 |
|  | Cogan, 2012 | Rb5 | 3.553 | 2.581 | 4.892 | 7.721 |
|  | Crowther, 2012 | Rb5 | 0.954 | 0.769 | 1.183 | 7.799 |
|  | Crowther, 2012 RCT | Rb5 | 1.250 | 0.143 | 10.940 | 4.237 |
|  | Hu, 2018 | Rb5 | 0.540 | 0.300 | 0.971 | 7.401 |
|  | Kabore, 2016 | Rb5 | 0.237 | 0.121 | 0.464 | 7.269 |
|  | Kalisa, 2017 | Rb5 | 0.531 | 0.111 | 2.535 | 5.445 |
|  | Kalok, 2018 | Rb5 | 1.675 | 0.479 | 5.853 | 6.122 |
|  | Kessous, 2013 | Rb5 | 5.300 | 0.622 | 45.180 | 4.285 |
|  | Kok, 2015 | Rb5 | 0.623 | 0.501 | 0.775 | 7.797 |
|  | Kugler, 2008 | Rb5 | 1.134 | 0.269 | 4.773 | 5.716 |
|  | Schemann, 2015 | Rb5 | 2.970 | 2.583 | 3.414 | 7.837 |
|  | Stattmiller, 2016 | Rb5 | 2.430 | 2.322 | 2.543 | 7.862 |
|  | Studsgaard, 2013 | Rb5 | 0.220 | 0.125 | 0.387 | 7.433 |
|  | Tsai, 2017 | Rb5 | 0.714 | 0.135 | 3.774 | 5.229 |
|  | Overall |  | *1.056* | *0.547* | *2.038* |  |
| Hypertension | Hammond, 2013 | Rb10 | 5.160 | 4.520 | 5.890 | 27.791 |
|  | Hu, 2018 | Rb5 | 2.200 | 1.588 | 3.047 | 27.352 |
|  | Kalisa, 2017 | Rb5 | 2.549 | 0.905 | 7.179 | 23.331 |
|  | Tsai, 2017 | Rb5 | 0.271 | 0.076 | 0.970 | 21.527 |
|  | Overall |  | *1.839* | *0.533* | *6.342* |  |
| Hysterectomy | Cheng, 2016 | Rb5 | 11.400 | 3.615 | 35.950 | 8.805 |
|  | Cogan, 2012 | Rb5 | 2.690 | 0.519 | 13.949 | 7.314 |
|  | Gilbert, 2012 | Rb5 | 4.009 | 1.130 | 14.218 | 8.454 |
|  | Homer, 2010 | Rb10 | 10.460 | 5.598 | 19.544 | 10.194 |
|  | Homer, 2010 | Rb5 | 3.950 | 2.189 | 7.127 | 10.268 |
|  | Homer, 2010 | Rb7 | 15.730 | 2.821 | 87.721 | 7.102 |
|  | Homer, 2010 | Rb8 | 11.570 | 2.712 | 49.368 | 7.895 |
|  | Kabore, 2016 | Rb5 | 0.354 | 0.047 | 2.694 | 6.242 |
|  | Kalisa, 2017 | Rb5 | 0.714 | 0.029 | 17.629 | 3.803 |
|  | Kugler, 2008 | Rb5 | 17.182 | 0.922 | 320.115 | 4.271 |
|  | Son, 2017 | Rb5 | 0.661 | 0.032 | 13.807 | 4.073 |
|  | Stattmiller, 2016 | Rb5 | 1.210 | 1.010 | 1.450 | 10.854 |
|  | Yao, 2017 | Rb5 | 0.998 | 0.729 | 1.367 | 10.725 |
|  | Overall |  | *3.390* | *1.562* | *7.356* |  |
| Infection | Crowther, 2012 | Rb5 | 1.490 | 0.333 | 6.674 | 10.137 |
|  | Gilbert, 2012 | Rb5 | 0.965 | 0.876 | 1.063 | 14.257 |
|  | Hu, 2018 | Rb5 | 0.500 | 0.117 | 2.136 | 10.322 |
|  | Kabore, 2016 | Rb5 | 1.746 | 1.059 | 2.879 | 13.660 |
|  | Kalisa, 2017 | Rb5 | 0.638 | 0.173 | 2.355 | 10.898 |
|  | Kugler, 2008 | Rb5 | 1.795 | 0.856 | 3.767 | 12.984 |
|  | Son, 2017 | Rb5 | 3.852 | 2.175 | 6.823 | 13.480 |
|  | Stattmiller, 2016 | Rb5 | 10.040 | 9.254 | 10.893 | 14.264 |
|  | Overall |  | *1.782* | *0.735* | *4.322* |  |
| Maternal Death | Kabore, 2016 | Rb5 | 0.206 | 0.028 | 1.526 | 43.553 |
|  | Motomura, 2017 | Rb5 | 4.450 | 1.149 | 17.240 | 56.447 |
|  | Overall |  | *1.168* | *0.153* | *8.930* |  |
| PE | Hammond, 2013 | Rb10 | 34.700 | 31.919 | 37.723 | 33.436 |
|  | Hu, 2018 | Rb5 | 1.240 | 0.791 | 1.943 | 32.293 |
|  | Kessous, 2013 | Rb5 | 0.200 | 0.019 | 2.145 | 16.541 |
|  | Tsai, 2017 | Rb5 | 1.156 | 0.127 | 10.539 | 17.729 |
|  | Overall |  | *2.759* | *0.711* | *10.713* |  |
| Placenta Previa | Asicioglu, 2014 | Rb5 | 1.640 | 1.331 | 2.021 | 53.339 |
|  | Baron, 2014 | Rb5 | 1.530 | 0.613 | 3.819 | 46.661 |
|  | Overall |  | *1.588* | *0.285* | *8.859* |  |
| Preterm Delivery | Hammond, 2013 | Rb10 | 6.700 | 6.297 | 7.128 | 100.000 |
|  | Overall |  | *6.700* | *0.642* | *69.927* |  |
| Retained Placenta | Belachew, 2014 | Rb5 | 1.790 | 1.651 | 1.941 | 50.012 |
|  | Kessous, 2013 | Rb5 | 0.570 | 0.518 | 0.627 | 49.988 |
|  | Overall |  | *1.010* | *0.192* | *5.308* |  |
| SMO | Crowther, 2012 | Rb5 | 0.668 | 0.433 | 1.030 | 20.452 |
|  | Litorp, 2016 | Rb5 | 0.950 | 0.489 | 1.846 | 19.581 |
|  | Mone, 2014 | Rb5 | 1.725 | 0.806 | 3.689 | 19.141 |
|  | Motomura, 2017 | Rb5 | 40.220 | 24.012 | 67.367 | 20.177 |
|  | Schemann, 2015 | Rb5 | 4.800 | 3.327 | 6.925 | 20.649 |
|  | Overall |  | *2.947* | *1.003* | *8.665* |  |
| Uterine dehiscence | Baron, 2014 | Rb10 | 2.780 | 2.060 | 3.751 | 23.193 |
|  | Baron, 2014 | Rb7 | 1.340 | 0.648 | 2.771 | 21.505 |
|  | Gilbert, 2012 | Rb5 | 2.009 | 1.120 | 3.601 | 22.195 |
|  | Kugler, 2008 | Rb5 | 53.016 | 3.141 | 894.724 | 9.610 |
|  | Stattmiller, 2016 | Rb5 | 0.800 | 0.702 | 0.912 | 23.498 |
|  | Overall |  | *2.190* | *0.702* | *6.836* |  |
| Uterine Rupture | Al-Zirqi, 2010 | Rb5 | 0.304 | 0.172 | 0.538 | 10.631 |
|  | Baron, 2014 | Rb5 | 2.650 | 0.620 | 11.333 | 8.132 |
|  | Cogan, 2012 | Rb5 | 0.352 | 0.071 | 1.757 | 7.660 |
|  | Jastrow, 2013 | Rb5 | 4.900 | 1.072 | 22.406 | 7.925 |
|  | Kabore, 2016 | Rb5 | 0.103 | 0.025 | 0.420 | 8.293 |
|  | Kalisa, 2017 | Rb5 | 0.192 | 0.011 | 3.497 | 4.445 |
|  | Kok, 2015 | Rb5 | 0.111 | 0.014 | 0.876 | 6.335 |
|  | Kugler, 2008 | Rb5 | 5.675 | 0.231 | 139.693 | 3.926 |
|  | Motomura, 2017 | Rb5 | 1.480 | 0.830 | 2.640 | 10.610 |
|  | Stattmiller, 2016 | Rb5 | 22.520 | 19.353 | 26.205 | 11.209 |
|  | Studsgaard, 2013 | Rb5 | 0.253 | 0.099 | 0.649 | 9.696 |
|  | Yao, 2017 | Rb5 | 0.118 | 0.093 | 0.150 | 11.139 |
|  | Overall |  | *0.730* | *0.333* | *1.604* |  |

STable 2

Subgroup Analysis The Correlation of Previous Caesarean Section and Adverse Maternal Outcomes

| RC | Outcomes | Studies | OR | | 95% CI | | Weight (%) |
| --- | --- | --- | --- | --- | --- | --- | --- |
|  |  |  |  |  | Lower Limit | Upper Limit |  |
| RC 5 | Analgesia/Anesthesia | **Crowther, 2012** | | 3.805 | 2.287 | 6.328 | 1.749 |
|  |  | **Crowther, 2012 RCT** | | 0.253 | 0.009 | 6.937 | 0.492 |
|  | Blood Transfusion | **Kok, 2015** | | 0.448 | 0.306 | 0.656 | 1.798 |
|  |  | **Yao, 2017** | | 0.792 | 0.697 | 0.900 | 1.857 |
|  |  | **Kessous, 2013** | | 0.900 | 0.402 | 2.012 | 1.601 |
|  | Heavy Bleeding | **Cogan, 2012** | | 3.553 | 2.581 | 4.892 | 1.817 |
|  |  | **Kabore, 2016** | | 0.237 | 0.121 | 0.464 | 1.673 |
|  |  | **Kalisa, 2017** | | 0.531 | 0.111 | 2.535 | 1.150 |
|  |  | **Kalok, 2018** | | 1.675 | 0.479 | 5.853 | 1.333 |
|  |  | **Kugler, 2008** | | 1.134 | 0.269 | 4.773 | 1.222 |
|  |  | **Schemann, 2015** | | 2.970 | 2.583 | 3.414 | 1.855 |
|  |  | **Studsgaard, 2013** | | 0.220 | 0.125 | 0.387 | 1.724 |
|  |  | **Tsai, 2017** | | 0.714 | 0.135 | 3.774 | 1.093 |
|  |  | **Belachew, 2014** | | 1.610 | 1.444 | 1.795 | 1.859 |
|  |  | **Hu, 2018** | | 0.540 | 0.300 | 0.971 | 1.714 |
|  |  | **Stattmiller, 2016** | | 2.430 | 2.322 | 2.543 | 1.864 |
|  |  | **Crowther, 2012** | | 0.954 | 0.769 | 1.183 | 1.843 |
|  |  | **Crowther, 2012 RCT** | | 1.250 | 0.143 | 10.940 | 0.848 |
|  |  | **Kok, 2015** | | 0.623 | 0.501 | 0.775 | 1.842 |
|  |  | **Kessous, 2013** | | 5.300 | 0.622 | 45.180 | 0.860 |
|  | Hypertension | **Kalisa, 2017** | | 2.549 | 0.905 | 7.179 | 1.465 |
|  |  | **Hu, 2018** | | 2.200 | 1.588 | 3.047 | 1.816 |
|  |  | **Tsai, 2017** | | 0.271 | 0.076 | 0.970 | 1.320 |
|  | Hysterectomy | **Cogan, 2012** | | 2.690 | 0.519 | 13.949 | 1.104 |
|  |  | **Kabore, 2016** | | 0.354 | 0.047 | 2.694 | 0.910 |
|  |  | **Kalisa, 2017** | | 0.714 | 0.029 | 17.629 | 0.515 |
|  |  | **Kugler, 2008** | | 17.182 | 0.922 | 320.115 | 0.587 |
|  |  | **Stattmiller, 2016** | | 1.210 | 1.010 | 1.450 | 1.849 |
|  |  | **Gilbert, 2012** | | 4.009 | 1.130 | 14.218 | 1.324 |
|  |  | **Son, 2017** | | 0.661 | 0.032 | 13.807 | 0.556 |
|  |  | **Homer, 2010** | | 3.950 | 2.189 | 7.127 | 1.713 |
|  |  | **Cheng, 2016** | | 11.400 | 3.615 | 35.950 | 1.396 |
|  |  | **Yao, 2017** | | 0.998 | 0.729 | 1.367 | 1.819 |
|  | Infection | **Kabore, 2016** | | 1.746 | 1.059 | 2.879 | 1.753 |
|  |  | **Kalisa, 2017** | | 0.638 | 0.173 | 2.355 | 1.300 |
|  |  | **Kugler, 2008** | | 1.795 | 0.856 | 3.767 | 1.636 |
|  |  | **Hu, 2018** | | 0.500 | 0.117 | 2.136 | 1.213 |
|  |  | **Stattmiller, 2016** | | 10.040 | 9.254 | 10.893 | 1.862 |
|  |  | **Gilbert, 2012** | | 0.965 | 0.876 | 1.063 | 1.860 |
|  |  | **Son, 2017** | | 3.852 | 2.175 | 6.823 | 1.721 |
|  |  | **Crowther, 2012** | | 1.490 | 0.333 | 6.674 | 1.186 |
|  | Maternal Death | **Kabore, 2016** | | 0.206 | 0.028 | 1.526 | 0.923 |
|  |  | **Motomura, 2017** | | 4.450 | 1.149 | 17.240 | 1.271 |
|  | PE | **Hu, 2018** | | 1.240 | 0.791 | 1.943 | 1.774 |
|  |  | **Tsai, 2017** | | 1.156 | 0.127 | 10.539 | 0.831 |
|  |  | **Kessous, 2013** | | 0.200 | 0.019 | 2.145 | 0.766 |
|  | Placenta Previa | **Asicioglu, 2014** | | 1.640 | 1.331 | 2.021 | 1.844 |
|  |  | **Baron, 2014** | | 1.530 | 0.613 | 3.819 | 1.537 |
|  | Retained Placenta | **Belachew, 2014** | | 1.790 | 1.651 | 1.941 | 1.862 |
|  |  | **Kessous, 2013** | | 0.570 | 0.518 | 0.627 | 1.860 |
|  | SMO | **Schemann, 2015** | | 4.800 | 3.327 | 6.925 | 1.803 |
|  |  | **Motomura, 2017** | | 40.220 | 24.012 | 67.367 | 1.746 |
|  |  | **Mone, 2014** | | 1.725 | 0.806 | 3.689 | 1.626 |
|  |  | **Litorp, 2016** | | 0.950 | 0.489 | 1.846 | 1.676 |
|  |  | **Crowther, 2012** | | 0.668 | 0.433 | 1.030 | 1.779 |
|  | Uterine dehiscence | **Kugler, 2008** | | 53.016 | 3.141 | 894.724 | 0.615 |
|  |  | **Stattmiller, 2016** | | 0.800 | 0.702 | 0.912 | 1.857 |
|  |  | **Gilbert, 2012** | | 2.009 | 1.120 | 3.601 | 1.716 |
|  | Uterine Rupture | **Cogan, 2012** | | 0.352 | 0.071 | 1.757 | 1.125 |
|  |  | **Kabore, 2016** | | 0.103 | 0.025 | 0.420 | 1.243 |
|  |  | **Kalisa, 2017** | | 0.192 | 0.011 | 3.497 | 0.593 |
|  |  | **Kugler, 2008** | | 5.675 | 0.231 | 139.693 | 0.516 |
|  |  | **Studsgaard, 2013** | | 0.253 | 0.099 | 0.649 | 1.522 |
|  |  | **Stattmiller, 2016** | | 22.520 | 19.353 | 26.205 | 1.854 |
|  |  | **Baron, 2014** | | 2.650 | 0.620 | 11.333 | 1.213 |
|  |  | **Al-Zirqi, 2010** | | 0.304 | 0.172 | 0.538 | 1.723 |
|  |  | **Jastrow, 2013** | | 4.900 | 1.072 | 22.406 | 1.174 |
|  |  | **Motomura, 2017** | | 1.480 | 0.830 | 2.640 | 1.718 |
|  |  | **Kok, 2015** | | 0.111 | 0.014 | 0.876 | 0.893 |
|  |  | **Yao, 2017** | | 0.118 | 0.093 | 0.150 | 1.838 |
|  |  | **Overall** | | 1.324 | 1.010 | 1.735 |  |
| Rc7 | Hysterectomy | **Homer, 2010** | | 15.730 | 2.821 | 87.721 | 39.303 |
|  | Uterine dehiscence | **Baron, 2014** | | 1.340 | 0.648 | 2.771 | 60.697 |
|  |  | **Overall** | | 3.528 | 0.681 | 18.269 |  |
| Rc8 | Hysterectomy | **Homer, 2010** | | 11.570 | 2.712 | 49.368 | 100.000 |
|  |  | **Overall** | | 11.570 | 0.992 | 134.911 |  |
| Rc10 | Hypertension | **Hammond, 2013** | | 5.160 | 4.520 | 5.890 | 20.401 |
|  | Hysterectomy | **Homer, 2010** | | 10.460 | 5.598 | 19.544 | 18.638 |
|  | PE | **Hammond, 2013** | | 34.700 | 31.919 | 37.723 | 20.455 |
|  | Preterm Delivery | **Hammond, 2013** | | 6.700 | 6.297 | 7.128 | 20.472 |
|  | Uterine dehiscence | **Baron, 2014** | | 2.780 | 2.060 | 3.751 | 20.034 |
|  |  | **Overall** | | 8.101 | 3.303 | 19.869 |  |

STable 3

Research Variables Operational Definition - Correlation between Previous CS and Adverse Maternal Outcomes

| Variable | Operational Definition | Parameter | Measuring Instrument | Scale |
| --- | --- | --- | --- | --- |
| *Independent* | | | | |
| Previous Caesarean | The surgical procedure in which incisions were made in the mother’s abdominal wall (laparotomy) and uterus (hysterectomy) to deliver one or more babies weighted >500grams | Robson Classification (RC) Group:  5. All multiparous women, with at least one previous uterine scar and a single cephalic pregnancy at greater than or equal to 37weeks gestation.  7. All multiparous women with a single breech pregnancy including, women with previous uterine scars.  8. All women with multiple pregnancies, including women with previous uterine scars  9. All women with a single pregnancy with a transverse or oblique lie, including women with previous uterine scars.  10. All women with a single cephalic pregnancy at less than or equal to 36weeks gestation, including women with previous scars. | Check list | Categorical |
| *Dependent* | | | | |
| Adverse maternal outcome | Unintended and unwanted case or condition that occurs after previous caesarean section during the subsequent pregnancy/childbirth, which is so detrimental to the health of a patient and can result in continuous harm that adjustment of therapy is needed | Analgesia/Anesthesia; Blood Tranfusion; Heavy Bleeding; Hypertension; Hysterectomy; Infection; Maternal Death; Pre-eclampsia; Placenta Previa; Preterm Delivery; Retained Placenta; Severe Maternal Outcomes; Uterine dehiscence; Uterine Rupture  ***Note:*** each outcome with statistical value (Odds Ratio or percentage with p-value) were gathered and analysed | data collection table | Ratio |
| *Confounding Variable* | | | | |
| Publication Year | The year when the studies being published | From 2008-2018 | data collection table | Integer |
| Countries | Location/setting where the study conducted | Countries all over the world | data collection table | Categorical |
| Study Design | The design used to conduct the study | Retrospective studies, Prospective studies, Randomized controlled trial studies | data collection table | Categorical |
| Sample size | part of the population to be studied or a portion of the characteristics of the population |  | data collection table | Numeric |

STable 4

Methodological quality assessment of the observational studies using the Newcastle Ottawa Scale

| Author | Adequate Definition of case | Non-exposed Cohort Selection | Non-exposed Cohort /Control Definition | Control for important factor | Exposure | Same method of ascertainment | Non response rate | Total |
| --- | --- | --- | --- | --- | --- | --- | --- | --- |
| Al-Zirqi, 2010 | * | * | * | ** | * | * |  | 7 |
| Asicioglu, 2014 | * | * | * | * |  | * |  | 5 |
| Baron, 2014 | * |  | * | * | * | * |  | 5 |
| Belachew, 2014 | * | * | * | * |  | * | * | 6 |
| Cheng, 2016 | * | * | * | * | * | * |  | 6 |
| Cogan, 2012 | * |  | * | * |  | * |  | 4 |
| Crowther, 2012 | * | * | * | ** | * | * |  | 7 |
| Gilbert, 2012 | * | * | * | ** | * | * |  | 7 |
| Hammond, 2013 | * | * | * | * | * | * |  | 6 |
| Homer, 2010 | * | * | * | * | * | * | * | 7 |
| Hu, 2018 | * | * | * | * | * | * |  | 6 |
| Jastrow, 2013 | * | * | * | * | * | * |  | 6 |
| Kabore, 2016 | * | * | * | * | * | * |  | 6 |
| Kalisa, 2017 | * | * | * | * | * | * |  | 6 |
| Kalok, 2018 | * | * | * | * | * | * |  | 6 |
| Kessous, 2013 | * | * | * | * | * | * |  | 6 |
| Kok, 2015 | * | * | * | * | * | * |  | 6 |
| Kugler, 2008 | * | * | * | ** | * | * | * | 8 |
| Litorp, 2016 | * | * | * |  | * | * |  | 5 |
| Mone, 2014 | * | * | * | * | * | * |  | 6 |
| Motomura, 2017 | * | * | * | ** | * | * |  | 7 |
| Schemann, 2015 | * | * | * | * | * | * |  | 6 |
| Son, 2017 | * | * | * |  | * | * |  | 5 |
| Stattmiller, 2016 | * | * | * | * | * | * | * | 7 |
| Studsgaard, 2013 | * | * | * | ** | * | * |  | 7 |
| Tsai, 2017 | * | * | * | ** | * | * | * | 8 |
| Yao, 2017 | * | * | * | * | * | * | * | 7 |


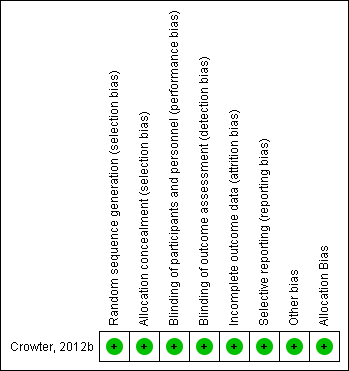


Figure A

Risk of bias assessed with Cochrane ROB tools ver.2.0 for Experimental Studies
